# Investigating treatment satisfaction and progress for offenders referred to community-based drug addiction treatment

**DOI:** 10.1186/1940-0640-10-S1-A75

**Published:** 2015-02-20

**Authors:** Yang Yang, Julie Gray, Jennifer Pankow, Patrick M Flynn, Kevin Knight

**Affiliations:** 1 Department of Psychology, University of Louisiana at Lafayette, Lafayette, LA, 70504, USA; 2 Institute of Behavioral Research, Texas Christian University, Fort Worth, TX, 76109, USA

## Background

The current study used a multilevel modeling technique to examine the influence of client-level factors and counselor-level variance on treatment satisfaction and progress for offenders referred to community-based drug addiction treatment.

## Materials and methods

The sample included 90 male participants (64 of which completed follow-up) and seven counselor participants (i.e., four females and three males) from community-based treatment in a Midwestern metropolitan area. Hierarchical linear modeling was conducted to examine the influence of victimization and violence history, psychiatric disorders (i.e., anxiety, depression), social functioning (i.e., social support, self-esteem), drug use severity, and treatment motivation on treatment satisfaction and progress after controlling for counselor-level variances. Hierarchical linear modeling also was employed to test the mediation of treatment satisfaction on the relationship between client-level factors and treatment progress.

## Results

Results indicated that higher levels of anxiety and depression were associated with a lower level of treatment satisfaction, and more social support was associated with increased satisfaction. Despite the non-significant relationship between treatment motivation and satisfaction, the influence of treatment motivation on treatment satisfaction was different across counselors. With regard to treatment progress, higher levels of depression predicted a decrease in progress, whereas more social support and treatment motivation were associated with an increased amount of treatment progress. Treatment satisfaction mediated the relationship of depression and social support with treatment progress, whereby a lower level of depression and more social support were associated with a higher level of treatment satisfaction, which in turn predicted a greater amount of client self-reported treatment progress.

## Conclusions and implications

The findings collectively underscore the importance of integrated interventions, social support, treatment motivation, and satisfaction on treatment outcome. Clinically, these findings emphasize the importance of: 1) incorporating psychological interventions into substance use treatment plans; 2) providing social support and increasing treatment motivation; and 3) enhancing treatment satisfaction in an effort to improve treatment outcome.

